# 

**Published:** 1852-10

**Authors:** 


### CONTENTS.

### JOURNAL.

Page.

ARTICLE I.—Treatment of Dental Caries, complicated with Disorders of the Pulp and Peridental Membrane. No. VI. By Robert Arthur, M. D., D. D. S. . . . . . . 3

ARTICLE II.—Essay on Professional Fees, read before the American Society of Dental Surgeons, August, 1852. By Elisha Townsend, M. D., D. D. S. . . . . . . . . . 18

ARTICLE III.—Proposition for Extending the Requirements of a Collegiate Dental Education. By B. Wood, M. D. . . . 28

ARTICLE IV.—From a Surgeon Dentist's Case Book. By J. L. Levison, D. D. S. . . . . . . . . . . 39

ARTICLE V.—A New Method of Supplying Artificial Teeth and Gums. By Wm. M. Hunter, Dentist, . . . . . 44

ARTICLE V.—Digestion and its Relation to the Teeth. By W. R. Handy, M. D. . . . . . . . . . . 57

ARTICLE VI.—Essay on Irregularity of the Teeth, read before the American Society of Dental Surgeons, August, 1852. Ey E. G. Tucker, M. D. . . . . . . . . . . 67

ARTICLE VII.—What can Dentists do to Advance their Profession? 73

ARTICLE VIII.—Eighth Annual Meeting of the Mississippi Valley Association of Dental Surgeons, . . . . . . 77

ARTICLE IX.—Hemorrhage from the Extraction of a Tooth. By S. A. SALTONSTALL, D. D. S. . . . . . . . 90

ARTICLE X.—Professional Merit. By S. D. Thompson, D. D. S. 91

ARTICLE XI.—Dissertation on the Effects of Carious Teeth, read before the American Society of Dental Surgeons, August 3, 1852. By Abr. Robertson, D. D. S. . . . . . . . . 93

ARTICLE XII.—Convulsions Cured by Lancing the Gums. By V. M. Swazye, D. D. S. . . . . . . . 102

### SELECTED ARTICLES.

ARTICLE XIII.—The sense of Taste and Effect of Plates worn in the Mouth. By W. H. Dwinelle, M. D. . . . . . 103

ARTICLE XIV.—The London Practice of Medicine contains a Cure, by Mr. Coote, of Inflammation and Abscess of the Tongue, 109

ARTICLE XV.—Cancrum Oris, . . . . . . . . . . 112

ARTICLE XVI.—Alveolar Hemorrhage Compress, constructed by Dr. R. Reid, Dentist, . . . . . . . . 117

*Contents.*

Article XVII.—The Jaws and Teeth in Semi-Barbarous Races of men, . . . . . . . . . . . . 121
Article XVIII.—Carcinomatous Tumor attached to the Uvula and Posterior Pillar of the Fauces; Removal and Recovery, 124
Article XIX.—Removal of one-half of the Lower Maxilla for Secondary Cancerous Manifestation, . . . . . . 127
Article XX.—Fissure of the Soft and Hard Palate; Operation. By Mr. Avery, . . . . . . . . . . . 129
Article XXI.—Observations on Excision of the Superior Maxillary Bone. By S. D. Gross, M. D. . . . . . 131
Article XXII.—Recurrence of an Osteo-cartilaginous Tumor, connected with the Nasal Process of the Superior Maxilla; Second Removal; Recovery. Under the care of M. Partridge, . 152

### BIBLIOGRAPHICAL.

Practical Observations on the Teeth, designed for Popular Use, . 154
A Review of Remarks on the Professional Education of Dentists, 154
Transactions Third Annual Meeting of the Medical Society of Ga. 155
A Lecture on Gun Shot Wounds, . . . . . . . 155
Difficulties and the Privileges of the Medical Profession, . . 155
New York Medical College Announcement. Session 1852-'3, . 155
Annual Catalogue and Announcement of the Medical Department of St. Louis University, . . . . . . . . . 156
Transactions Medical Association of Missouri, . . . . . 156
Transactions of the Medical Society of Pennsylvania, . . . 156
Physicians' Pocket Visiting List, Diary and Almanac for 1853, . 156
Second Annual Announcement New York College Dental Surgery, 156
Principles and Practice of Dental Surgery, . . . . . 157
A Practical Treatise on Dental Medicine, . . . . . . 157

### EDITORIAL DEPARTMENT.

American Society of Dental Surgeons, . . . . . . 158
New Method of Treating Teeth after the Lining Membrane has become Exposed, . - . . . . . . . 160
Fauchard, . . . . . . . . . . . . . 162
Dental Exostosis, . . . . . . . . . . . 162
Dental Works in Preparation for the Press, . . . . . . 163
Effects of Tobacco, . . . . . . . . . . . 163
Singular Death, . . . . . . . . . . . . 164
Muscular Power, . . . . . . . . . . . 165
Necrosis of the Superior and Inferior Maxilla, . . . . . 166
Live and Learn, . . . . . . . . . . . 167
Prize Essays, . . . . . . . . . . . . 168

